# Partial vaccination and associated factors among children aged 12–23 months in eastern Ethiopia

**DOI:** 10.1186/s12887-022-03320-3

**Published:** 2022-05-12

**Authors:** Menberu Muluye, Lemessa Oljira, Addis Eyeberu, Tamirat Getachew, Adera Debella, Alemayehu Deressa, Merga Dheresa

**Affiliations:** 1 Haramaya Health Office in Haramaya, East Hararge Zone, Oromia region, Harar, Ethiopia; 2 School of Public Health, College of Health and Medical Sciences, Haramaya University, Harar, Ethiopia; 3 School of Nursing and Midwifery, College of Health and Medical Sciences, Haramaya University, Harar, Ethiopia

**Keywords:** Partial immunization, Vaccination status, Children, Predictors, Haramaya

## Abstract

**Background:**

Vaccine prevents about 2–3 million deaths from vaccine-preventable diseases each year. However, immunization coverage in Ethiopia is lower than the herd immunity level required to prevent the spread of all vaccine-preventable diseases. Thus, this study aimed to assess the partial immunization and associated factors among 12–23-month-old children in Eastern Ethiopia.

**Method:**

A community-based cross-sectional study design was carried out among 874 randomly selected mothers/caregivers of children aged 12–23 months. A structured questionnaire was adapted and data were collected through face-to-face interviews and review of vaccination cards. Data were coded and analyzed using the Stata version 14 software. A binary logistic regression model was utilized to identify the determinant factors. The predictor of partial immunization was presented by an adjusted odds ratio with a 95% confidence interval. A *p*-value of < 0.05 was used to establish statistical significance.

**Result:**

The prevalence of partial immunization was 31.4% (95% CI: 28–35). The dropout rate between the first and third pentavalent vaccine was 17%. Being female child [AOR = 0.73, 95% CI: 0.52–0.95], 18–20 month child [AOR = 1.6, 95% CI: 1.1- 2.4], the child born to mothers who heard about vaccination [AOR = 3.9, 95%CI: 1.92- 8.01], a child born to mother who did not receive immunization counselling [AOR = 1.65, 95%CI: 1.15–2.36], and child whose mother walk 15–30 min, 31–60 min, and > 60 min to reach nearby health facilities [AOR = 1.94, 95% CI: 1.1–3.45], [AOR = 4.5, 95% CI: 2.47–8.15], and [AOR = 3.45, 95% CI: 1.59- 7.48] respectively were factors significantly associated with partial vaccination.

**Conclusions:**

The prevalence of partial immunization is high compared to other studies. As a result, to decrease the proportion of defaulters and to increase immunization coverage, maternal health care utilization like antenatal care follow-up and mother knowledge about the importance of the vaccine need to be sought cautiously.

## Introduction

Immunization is the most important and cost-effective public health intervention. Reducing infant mortality and morbidity caused by infectious diseases is an important strategy [1]. Vaccination saves the lives of about 2 to 3 million children each year. However, in developing countries, 8 million children die from vaccine-preventable illnesses before the age of one [2, 3].

Despite improved vaccination performance [4, 5], approximately 23 million children had not had adequate access to the vaccine in 2020 [6]. About 60% of these children lived in low- and middle-income countries [7]. Almost one in five African children is not fully vaccinated [8]. Similarly, in many Ethiopian regions, the immunization coverage is less than 80% and the expanded vaccination program (EPI) schedule is not completed as planned [9]. According to a recent report from Ethiopia, only 43% of children aged 12 to 23 months were completely vaccinated and 19% were unvaccinated [10]. In addition, another study found that 46 percent of children were only partially immunized [11].

Factors such as children born in a health facility, born to 30–39 years old, attending at least four visits, and having high-income mothers [12–14] were significantly associated with partial vaccination. Furthermore, lack of access to immunization services, long distance to health facilities, lack of knowledge about immunization, have no faith in immunization, fear of side effects, and lack of transportation to health facilities was the main reason for incomplete vaccination [13, 15].

To address the issue of partial immunization and low immunization coverage, the world health organization has endorsed the immunization agenda 2030, a new global vision and strategy (IA2030) [7]. Similarly, Ethiopia has deployed and strengthened the women's development army (WDA) initiative as part of the health extension program package to improve access to and provision of immunization services [16, 17].

Despite the implementation of several strategies by the government and other stakeholders aimed at improving immunization coverage, the number of fully vaccinated children remains far below the target [10]. The country's frequent measles outbreaks and high child morbidity and mortality rates may be attributed to low immunization coverage. Around 40% of Ethiopian children do not benefit from immunization [18] and similar evidence showed that 26.8% of children were at risk of acquiring vaccine-preventable diseases [2].

To improve child immunization coverage, the determinants of partial vaccination need to be investigated [16]. Furthermore, information on the prevalence of partial immunization among children aged 12–23 months in eastern Ethiopia is scarce. As a result, this study was aimed to assess the partial immunization and associated factors among children aged 12–23 months in Haramaya district, eastern Ethiopia.

## Methods and materials

### Study design, area, and period

A community-based cross-sectional study was conducted in Haramaya District, East Hararge zone; Oromia Region in Ethiopia from January 1^st^ to January 30, 2021. The district is 506 km away from Addis Ababa, the capital of Ethiopia. Haramaya district has 2 urban and 32 rural kebeles. According to the 2007 national census, the total population of Haramaya district is 304, 849; of which 152,119(49.9%) and 152,729(51.01%) were males and females respectively; with 9,816 (3.22%) 12 to 23-month-old children. There are 8 health centers, 38 health posts, and 13 low-level private clinics in the district (Haramaya administrative health office report for 2018).

### Populations

Mothers/caregivers with children aged between 12 to23 months who lived in Haramaya district during the study period comprised the study population. Mothers who had an alive child aged between 12–23 months and lived in the area were eligible.

### Sample size determination and sampling procedure

The sample size was calculated by using a single population proportion formula with assumptions of confidence level at 95% = 1.96, a margin of error (d) = 0.03, and a proportion of children aged 12–23 months with immunization coverage (*P* = 0.383) was taken from a study conducted by Tamirat, K.S and Sisay M.M [14] and by adding 5% non-response rate and design effect 1.5, the final sample size became 892.

From 34 kebeles of Haramaya district, 5 of them were selected randomly. There were 7675 households in those five kebeles. The calculated sample size (892) was then allocated proportionally to the selected kebeles based on their population (number of mothers/caregivers). Individual study participants were chosen at random from each kebele using a simple random sampling technique. For households with more than one eligible member, an interview was conducted by selecting one woman through a lottery method.

### Data collection method and Quality control

The data were collected using a structured questionnaire adapted from the Ethiopian demographic health survey (EDHS [19] and previous literature [20]. It is divided into five sections: socio-demographic data, vaccination knowledge, maternal health care utilization, access and quality of vaccination services, and child vaccination. The questionnaire was written in English, translated to the local languages (Afan Oromo and Amharic) in the study area, and then translated back to English to ensure consistency. Five nurses collected data through face-to-face interviews. Vaccination data were collected from the child’s immunization card, or through an interview if the immunization card was not accessible. Where vaccination card was not accessible, confirmation was done by observing BCG scar. Data collectors and supervisors were trained on how to ask and fill questions, how to select households and children, and how to approach mothers/caregivers. Before the actual data collection, the questionnaire was pre-tested on 5% of non-selected households. The completeness of filled questionnaires was verified.

### Operational definition

#### Partially vaccinated

Twelve to twenty-three months old child who received at least one vaccine, but not all the EPI vaccines. In this study, those children who belong to defaulters/do not belong to fully vaccinated were leveled as partially vaccinated were as those children not belongs to the above definition were leveled as not partially vaccinated.

#### Fully vaccinated

A 12–23 months old child who received one dose of BCG and measles, three doses each of the Pentavalent, four doses of OPV, three doses of PCV, and two doses of Rota vaccine before his/her first birthday.

#### Unvaccinated

A 12–23-month-old child who did not receive any of the EPI vaccines.

##### Immunization coverage by card

 The vaccination coverage calculated with numerator based only on mothers/caregivers’ reports.

##### Immunization coverage levels

Represent the percentage of a target population that has been vaccinated. Coverage is usually calculated for each vaccine and the number of doses received. It is, therefore, the percentage of children within the target population who received vaccinations against specific vaccine-preventable diseases by a certain age and who were reported and documented.

##### Dropout rate (DOR)

Is the rate difference between the initial vaccines (BCG or Pentavalent I) and the final vaccines (Pentavalent III or Measles).

BCG to Measles dropout rate: the percent of children vaccinated for BCG who don’t receive measles vaccine.

Pentavalent I to Pentavalent III dropout rate: the percent of children vaccinated for Pentavalent I, but who did not receive Pentavalent III.

Knowledge of mothers/ caregivers on immunization were measured through 5 knowledge-related questions and the correct answer was level as 1 and the incorrect answer is leveled as 0 and the result was described.

### Statical analysis

The data were coded, cleaned, edited, and entered into Epi data statistical software version 3.1 and then exported to STATA version 14 for analysis. Summary statistics were presented with percentages, mean, standard deviation, median and interquartile range. Binary logistic regression was used to find out predictors of vaccination status. The outcome variable was dichotomized into “Yes (partially vaccinated)” and “No (not partially vaccinated)”.

Bivariate analysis and multivariate analysis were done to see the association between each independent variable and partial vaccination by using binary logistic regression. Variables with *p*-values less than 0.25 in the bivariable analysis were selected for further inclusion in the multivariable model. The multi-co-linearity test was carried out to see the correlation between independent variables by using the standard error (standard error > 2 was considered as suggestive of the existence of multi-co-linearity). The association between outcome and predictors was reported by AOR with a 95% confidence interval. *P*-value less than 0.05 was considered as a cut-off point for statistical significance. Likewise, after fitting the model goodness of the final model was checked by using the Hosmer- Lemeshow test. The Hosmer–Lemeshow statistic indicates a good fit at a *p*-value of 0.05 or greater.

### Ethical consideration

Ethical clearance to conduct this study was obtained from Haramaya University, College of Health and Medical Sciences, Institutional Health Research Ethics Review Committee (HU-IHRERC). A permission letter was obtained from the district administration and district health office. Informed, voluntary, written, and signed consent was obtained from individuals that were going to be involved in the study, following an explanation about the purpose of the study, risk, and benefit. Confidentiality was kept throughout the data collection and the entire study period. The right to participate or not to participate in the study was explained to the participants.

## Result

### Socio-demographic characteristics of the study participants

A total of 874 mothers/caregivers who had 12‒23 month-old children participated in the study with a response rate of 98%. The majority of the respondents were between the ages of 25 and 34 (44.2%), Oromo (98.5%), and married (89.2%). In this study, the average age of children was 16 months, and more than half 462(52.9%) of the participants were male. More than half of the mothers (67.9%) were unable to read and write (Table 1).

**Table 1:** Socio-demographic characteristics of the mothers/caregivers in Haramaya district, East Hararge zone, Eastern Ethiopia, 2021(*n* = 874)

Variable	Frequency	Percentage (%)	%Not Vaccinated	%Partially Vaccinated	%Fully Vaccinated
Mother’s age(year)					
15–24	289	33.0	36.3	32.8	32.1
25–34	386	44.2	37.0	43.8	47.0
35–45	199	22.8	26.7	23.4	20.9
Marital status of the mother/caregiver					
Single	10	1.1	10.0	20.0	70.0
Married	780	89.2	17.1	31.8	51.2
Separated	42	4.8	31.0	31.0	38.0
Divorced	17	2.0	23.5	17.7	58.8
Widowed	25	2.9	24.0	32.0	44.0
Occupation status					
House wife	422	48.3	64.3	48.9	42.2
Farmer	312	35.7	28.6	43.1	33.6
Government employee	31	3.5	1.3	2.2	5.2
Merchant	97	11.1	4.5	5.1	17.2
Daily labour	12	1.4	1.3	0.7	1.8
Educational level					
Unable to read and write	593	67.9	91.1	73.7	55.9
Read and write	160	18.3	7.1	19.3	21.7
Grade 1–8	91	10.4	0.6	4.7	17.4
Grade 9–12	17	1.9	0.6	1.9	2.5
College/university	13	1.5	0.6	0.4	2.5
Religion					
Muslim	848	97.0	0.6	1.1	4.1
Christian	26	3	99.4	98.9	95.9
Monthly income					
Less than 2000	10	1.1	0.6	0.7	1.6
2000–3000	31	3.6	3.8	2.6	4.1
3001–4000	47	5.4	3.8	4.4	6.5
4001–5000	84	9.6	27.4	11.3	10.6
Greater than 5000	331	37.9	27.4	33.6	44.2
I do not know	371	42.5	60.5	47.4	32.9
Age of child					
12-14montths	300	34.3	33.8	27.7	38.6
15-17 months	257	29.4	26.1	29.2	30.7
18-20 months	212	24.3	24.8	29.2	21.0
21-23 months	105	12.0	15.3	13.9	9.7
Sex of child					
Male	462	52.9	57.3	56.9	48.8
Female	412	47.1	42.7	43.1	51.2

### Knowledge of the mother/ caregivers on immunization

Almost all 803 (91.9%) of the participants have heard about immunization as a program. More than half (56.1%) of the caregivers heard a message about the importance of vaccination. Thirty (3.4%) of the participant knew where to get regular vaccinations. Two hundred sixty-seven (30.5%), 10 (1.1%), and 6 (0.7%) knew when to return to the next vaccination from the campaign and when to get regular vaccinations respectively.

One hundred ninety-four (24.2%) of mothers believe that vaccination is essential for disease prevention. Regarding the mother's understanding of the age at which the child begins and ends vaccination, 31.5% and 28.6% of mothers knew the age at which the child should start or complete vaccination respectively (Table 2).

**Table 2:** Knowledge of mothers/caregivers on vaccination at Haramaya district, East Hararge, Eastern Ethiopia, 2021

Variable	Frequency	Percentage (%)	%Not Vaccinated	%Partially Vaccinated	%Fully Vaccinated
heard about vaccination (*n* = 874)					
No	71	8.1	65.0	95.6	99.1
Yes	803	91.9	35.0	4.4	0.9
Source of information (*n* = 803)					
community members	63	7.9	21.4	10.8	5.9
Health care workers	307	38.2	27.2	37.6	40.3
Health extension workers	299	37.2	32.0	38.2	35.9
TV/Radio/Newspaper	134	16.7	19.4	13.4	17.9
Message content (*n* = 803)					
About campaign	267	30.5	24.3	45.7	30.0
Importance of vaccination	490	56.1	6.7	25.5	67.8
Where to get vaccination	30	3.4	6.7	30.0	63.3
Age to get vaccination	6	0.7	0.0	33.3	66.7
When to return for the next doses	10	1.1	20.0	40.0	40.0
The benefit of vaccinating children (*n* = 803)					
To prevent the diseases	194	24.2	5.7	26.3	68.0
For specific diseases	90	11.2	0.0	30.0	70.0
For child health	276	34.4	6.5	27.2	66.3
Others	10	1.2	0.0	20.0	80.0
I do not know	233	29.0	41.9	39.2	18.9
Age Starting vaccination (*n* = 803)					
Just after birth	253	31.5	5.1	17.2	44.7
Four weeks after birth	158	19.7	5.7	14.2	24.8
Six weeks after birth	114	14.2	3.2	17.2	14.0
I do not know	278	34.6	86.0	51.4	16.5
Sessions needed for full vaccination (*n* = 803)					
Correct session	364	45.3	13.0	34.0	62.7
Incorrect session	439	54.7	87.0	66.0	37.3
Age to completed vaccination(*n* = 803)					
< 1 year	230	28.6	3.0	21.4	75.6
> 1 year	273	34	4.4	26.4	69.2
I do not know	300	37.4	37.0	41.1	21.9
Vaccination may cause health problem					
No	738	84.4	14.0	30.0	56.0
Yes	36	4.1	63.9	19.4	16.7
I do not know	100	11.4	30.0	46.0	24.0
Mother/caregiver ever decided the child to get the vaccine(*n* = 874)					
No	797	91.2	12.8	32.2	55.0
Yes	77	8.8	71.4	22.1	6.5

### Accessibility and quality of vaccination services

Almost all 871 (99.7%) participants stated that they have access to health facilities that provide immunization services. More than half of (57%) participants receive advice from a health worker about the importance of vaccines, their side effects, and the completion of child vaccinations. However, 376 (43%) of mothers/caregivers did not receive counseling services. Among study participants, 392 (44.9%) said they were satisfied with the vaccination service. Among mothers who take their children for vaccination, 482 (55.1%) said they were refused the service due to the service provider's medium and poor approaches (Table 3).

**Table 3:** Access and quality of vaccination in Haramaya District, Eastern Ethiopia, 2021

Variable	Frequency	Percentage %	%Not Vaccinated	%Partially Vaccinated	%Fully Vaccinated
Nearby health facility with vaccination (*n* = 874)					
No	3	0.3	1.3	0.4	0.0
Yes	871	99.7	98.7	99.6	100.0
Health facilities (*n* = 874)					
Hospital	9	0.01	0.7	0.7	1.4
Health center	252	20.8	12.1	20.1	40.0
Health post	601	68.8	83.4	78.5	57.6
Private clinic	12	1.4	3.8	0.7	1.0
Means of transportation to the health facility(*n* = 874)					
Walk	607	69.5	79.6	75.9	61.8
Any means of transportation	267	30.5	20.4	24.1	38.2
Time required to reach the nearby health Facility(*n* = 874)					
< 15 min	119	13.6	4.5	6.2	21.4
15–30 min	386	44.2	21.0	34.7	58.2
31-60 min	305	34.9	57.3	50.7	17.2
> 60 min	64	7.3	17.2	8.4	3.2
Health worker advice about vaccination(*n* = 874)					
Yes	498	57.0	92.4	54.7	18.3
No	376	43.0	7.6	45.3	81.7
Long waiting line(*n* = 874)					
Yes	453	51.8	79.0	58.4	38.2
No	421	48.2	21.0	41.6	61.8
Satisfaction of vaccination services(*n* = 874)					
Good	392	44.9	15.9	37.6	59.6
Medium	402	46.0	52.9	53.7	38.8
Bad	64	7.3	24.2	7.3	1.4
I do not know	16	1.8	7.0	1.4	0.2

### Partial vaccination

The prevalence of partial immunization was 31.4% (95% CI: 28–35). The full immunization coverage was 50.7% (95% CI: 47–54). One hundred fifty-seven (18.0%) of the children had received no vaccine at all. Polio-1 (81.5%) and PCV-3 (55.5%) had the highest and lowest vaccine coverage respectively. The majority of the children (82.0%) received the vaccination, and 470 (65.6%) of mothers displayed the child vaccination card (Table 4).

**Table 4:** Vaccination coverage among children aged 12–23 months by card plus history in Haramaya district, East Hararge zone, Eastern Ethiopia, 2021

Variable	Frequency	Percentage (%)	%Not Vaccinated	%Partially Vaccinated	%Fully Vaccinated
Vaccinated (partially & fully vaccinated) *n* = 874					
Yes	717	82.0	0.00	100.0	100.0
No	157	18.0	100.0	0	0
Vaccination card(*n* = 717)					
Yes	470	65.6	0	36.5	83.5
No	247	34.4	0	63.5	16.5
Place of vaccination (717)					
At home	60	8.4	0	19.0	1.8
During campaign	88	12.3	0	28.5	2.3
The nearest health institution	489	68.2	0	35.0	88.7
Out reach	80	11.2	0	17.5	7.2

**Fig. 1:**
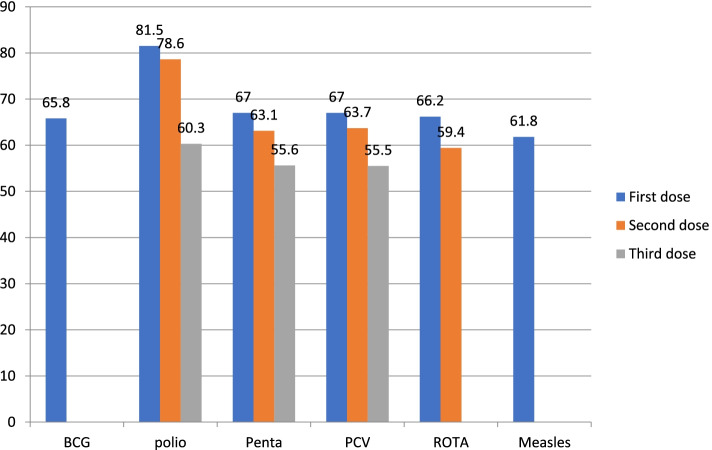
Vaccination coverage among children aged 12–23 months in Haramaya district, eastern Ethiopia, 2021

More than half of the children were immunized against BCG (65.8% [95%ci: 62.6%, 68.9%], OPV-3 (60.3% [85%CI: 57%, 63.5%], Pentavalent-3 (55.6% [95% CI: 52.3%, 58.9%], PCV3 (55.5% [95% CI: 52.2%, 58.8%], ROTA2 (59.4% [95% (Fig. 1). The drop-out rate of pentavalent 1 and 3 vaccine were a 17%. While the drop-out rate for Pentavalent-1 and measles was 7.8%.

### Factors associated with partial vaccination

In the final model of multi-variable binary logistic regression analysis, being female child, being 18–20 months old child, the child born to mothers who don’t hear about vaccination, a child born to mother who did not receive immunization counseling, and child whose mother walk 15–30 min, 31–60 min, and > 60 min to reach nearby health facilities respectively were all factors significantly associated with partial vaccination. Female children have 27% lower odds of being partially vaccinated than male children [AOR = 0.73, 95% CI: 0.52–0.95]. The odds of being partially vaccinated among children aged 18–20 months was 1.6 times higher than those children aged 12–24 months [AOR = 1.6, 95% CI: 1.1- 2.4]. The odds of being partially vaccinated among children whose mothers did not hear about vaccination was 3.9 times higher than their counterparts [AOR = 3.9, 95%CI: 1.92- 8.01]. The odds of being partially vaccinated was 1.65 times higher among children whose mothers had not received counseling about vaccination [AOR = 1.65, 95%CI: 1.15–2.36].

The odds of being partially vaccinated was 1.94, 4.5, and 3.45 times higher among children whose families traveled 15–30 min, 31–60 min, and more than 60 min to reach nearby health facilities compared to children whose family traveled less than 15 min [AOR = 1.94, 95% CI: 1.1–3.45], [AOR = 4.5, 95% CI: 2.47–8.15], and [AOR = 3.45, 95% CI: 1.59- 7.48] respectively (Table 5).

**Table 5:** Factors associated with partial vaccination status among children of 12 to 23 months of age in Haramaya district, eastern Ethiopia, 2021

Variables	Categories	Partial vaccination		COR (95%CI)	AOR (95%CI)
		No	Yes		
Age of child (in months)	12–14	224	76	1	1
	15–17	177	80	1.3(0.92–1.92)	1.2(0.84–1.8)
	18–20	132	80	1.79(1.22–2.6)	1.6(1.1- 2.4) **
	21–23	67	38	1.67(1.03–2.68)	1.5(0.91- 2.5)
Sex of child	Male	306	156	1	1
	Female	294	118	0.78(0.59–1.04)	0.73(0.52–0.95)
Mother’s Age (years)	15–24	199	90	1	1
	25–34	266	120	0.99(0.72–1.38)	0.96(0.68–1.36)
	Above 35	135	64	1.04(0.7–1.54)	0.99(0.66–1.5)
Heard about vaccination	Yes	59	12	1	**1**
	No	541	262	2.38(1.3–4.5)	3.9(1.92- 8.01) **
ANC Visit	No	521	244	0.81(0.52–1.27)	0.67(0.39–1.14)
	Yes	79	30	1	1
Place of delivery	Health facility	377	147	1	**1**
	Home	223	127	1.46(1.1–1.95)	1.1(0.76–1.56)
Time took to reach the nearby health facility	< 15 min	102	17	1	1
	15–30 min	291	95	1.96(1.12–3.44)	1.94(1.1–3.45) *
	31–60 min	166	139	5.02(2.87–8.8)	4.5(2.47–8.15) *
	> 60 min	41	23	3.37(1.6–6.94)	3.45(1.59- 7.48) *
Counselling about vaccination	No	374	124	2.01(1.50–2.67)	1.65(1.15–2.36) **
	Yes	226	150	1	1

Abbreviation: *AOR* Adjusted Odds Ratio, *COR* Crude Odds Ratio, *CI* Confidence Interval

*p*-value * < 0.05, ** < 0.01, *** < 0.001, 1 = reference

## Discussion

This study assessed the prevalence of partial vaccination among children aged 12–23 months old. Furthermore, factors associated with partial vaccination were identified.

This study showed that the proportion of children who were partially vaccinated was 31.4% (95% CI: 2835). This finding was higher than studies conducted in the city of Arba Minch in southern Ethiopia [21] and the city of Woldiya in northern Ethiopia 11.4% [22]. This disparity could be explained by differences in sample size and socio-demographic characteristics. The prevalence of fully vaccinated children at age of 12–23 was 50.6% (95 CI: 47, 54). This is consistent with research conducted in Oromia regional state, eastern Ethiopia 52.9% [23]. However, the finding of this study is lower than those of previous studies in Jigjig, Ethiopia 74.6% [24], Woldiya, Ethiopia 87.7% [22], and Ghana 89.5% [25]. These disparities could be due to differences in the availability and accessibility of services. This low coverage implies that there is a significant gap in the provision of health information and the utilization of health services by caregivers/mothers. The use of health services such as ANC should be increased because it is the best place for mothers to be counseled on the importance of vaccination.

In this study, the odds of a female child being partially vaccinated is reduced by 27% compared to a male child. This finding is consistent with a study conducted in Ghana [26] and Nigeria [27]. This could be attributed to similarities in study settings as well as sociodemographic characteristics of the study participants. This study also revealed that a child's age was related to partial immunization. An 18–20-month-old child is 60% more likely to be partially vaccinated than a 12–14-month-old child. A possible justification could be the mother's or caregiver's perception that the vaccine's importance diminishes as the child grows older [28].

Finding from this study pointed out that children of mothers who did not hear about vaccination were more likely to be partially vaccinated than their counterparts. The possible justification is that mothers who did not receive information or were not exposed to vaccination may not know the significance of vaccination, resulting in a lack of knowledge and, as a result, their children may be partially vaccinated. Children of unvaccinated mothers were more likely to be partially vaccinated, according to the evidence [11].

According to the findings of this study, children whose mothers had to walk 15–30 min, 31–60 min, or more than 60 min to reach a nearby health facility were more likely to be partially vaccinated than those whose mothers had walked less than 15 min. This finding contradicts studies conducted in Togo [13] and Nigeria [29]. One possible explanation is that roads are in poor condition, resulting in a lack of transportation (vehicle) to reach health facilities. Evidence suggests that improving immunization coverage necessitates strengthening outreach strategies [13].

In this study, immunization counseling was found to be significantly associated with partial immunization. A child born to a mother who did not receive immunization counseling was more likely to be partially vaccinated than a child born to a mother who did receive immunization counseling. One possible explanation is a lack of utilization of healthcare services, which results in the absence of services such as immunization counseling. Evidence suggests that poor maternal service utilization is significantly associated with partial immunization [30]. Strengthening service utilization could avert the problems of partial vaccination.

## Conclusion

The prevalence of partial vaccination was higher than studies done in Ethiopia. Furthermore, low vaccine coverage compared to the national EPI coverage plan (75%) was discovered in this investigation. In the study, being a female child, being 18–20 months old child, the child born to mothers who did not hear about vaccination, a child born to a mother who did not receive immunization counseling, and child whose mother walks 15–30 min, 31–60 min, and > 60 min to reach nearby health facilities respectively were factors significantly associated with partial vaccination. As a result, maternal health care utilization should be advocated to reduce the proportion of partial vaccination.

## Data Availability

This study includes all relevant data. However, on reasonable request, the corresponding author will provide additional data.

## Abbreviations

ANC: Antenatal Care
BCG: Bacilli Calmette Guerin
CRRR: Crude Relative Risk Ratio
DOR: Dropout Rate
DPT: Diphtheria Pertussis Tetanus
EPI: Expanded Programme on Immunization
PCV: Pneumococcal Conjugate Vaccine
VPDs: Vaccine-Preventable Diseases
WHO: World Health Organization
